# Thanks to reviewers list 2025

**DOI:** 10.1080/0886022X.2025.2600690

**Published:** 2026-01-04

Zaid Abassi

Dina A Abdellatif

Aly M. Abdelrahman

Abdollah Abdollahpour

Santibuana Abd Rahman

Thiruvaimozhi Abimannan

Mohamed Aboelnasr

Remon Abu-Elyazeed

Benjamin A. Adam

Adebowale Adebiyi

Deborah B Adey

Sumarno Adi Subrata

Esra Adiyeke

Frank Adusei-Mensah

Mohamed A. El-Hefny

Hanri Afghahi

Anurag Agarwal

Ana De Lurdes Agostinho Cabrita

Madhu Sudan Agrawal

Zhilong Ai

Sebnem Senol Akar

Oleh Akchurin

Nazife Akman

Salma Aktar

Gulali Aktas

Fadhl M. Alakwaa

Abdallah Alami

Amin Alamin

Rehab Albakr

Ebraheem Albazee

Roberto Alcázar Arroyo

Tim Alefantis

Akehu Alemasi

Suceena Alexander

Abdullah Alhwiesh

Ahlam Ali

Hatem Ali

Abdullah Alkattan

Hayder Al-Kuraishy

Ali Abdulmajid Dyab Allawi

Mohammad Allibaih

Marco Allinovi

Fadel Alrowaie

Alanoud Alshami

Osama Alshogran

Mehmet Altintas

Ahmet Alver

Mohammed Al-Zobaidy

Cristián A. Amador

Prince Keshide Amaechi

Justin Z Amarin

Daniela Amicizia

Floriano Amimo

Mohsen Amin

Morteza Amirteimoury

Emanuele Amodio

Afolake Amodu

Porntep Amornritvanich

Edina Amponsah-Dacosta

Deeba Amraiz

Won Suk An

Xingyue An

Anand Anbarasu

Austin Anderson

Theodoros Androutsakos

Germán Áñez

Gary Ang

Andrea Angeletti

Guangyu Ao

Seyyed Mostafa Arabi

Samsul Arefin

Jesu Arockiaraj

Puneet Arora

Eliott Arroyo

Mustafa Arslan

Ergin Arslanoglu

Ayşe Serra Artan

Ferruh Artunc

Asmik Asatryan

Milad Ashrafizadeh

Mahendra Kumar Atlani

Filippo Aucella

Jagannadha Avasarala

Ivica Avbersek-Luznik

Petar Avramovski

Yavuz Ayar

Aryan Ayati

Zehra Aydin

Nazar Mohd Zabadi Mohd Azahar

Daniel Azevedo Mendes

Kemal Ağbaht

Carla C. Baan

Janka Bábíčková

Dalia M. Badary

Gamal Badr

Sangrak Bae

Jiang Bai

Mi Bai

Peng Bai

Qiong Bai

Nazia Begum Bakerally

Austin Baldwin

Ismail Baloğlu

Manish R Balwani

Santasree Banerjee

Christos Bantis

Hao Bao

Kun Bao

Allan John R. Barcena

Simge Bardak DemİR

Ivona Baricevic-Jones

Jonatan Barrera Chimal

Nicholas Barrett

Guillermina Barril

Chris Bartlett

Maria Bartosova

Abu Bashar

A. Basher

Gulsum Iclal Bayhan

Deniz Bayraktar

Jagadeesh Bayry

Sonam Bedi

Robert Bednarczyk

Andrew Beenken

Justin M. Belcher

Julie Belliere

Jerzy Beltowski

Bright Benfor

Youssef Bennis

Alexander Berezin

Elke Bergmann-Leitner

Philippe Beutels

Sebastjan Bevc

Mehmet Beyazal

Rodrigo Bezerra

Nataliya Bgatova

Joyita Bharati

Jennifer B. Harris

Omesh Bharti

Sonu Bhaskar

Rohan Bhattacharya

Sankha Bhattacharya

Dipankar Bhowmik

Prakash Bhuyan

Xiao Bi

Aida Bianco

Luigi Roberto Biasio

Yohan Bignon

Ayse Bilgic

Satılmış Bilgin

Mark Blatter

Alon Bnaya

Dannell Boatman

Mickaël Bobot

Margherita Bodini

Maristela Bohlke

Maristela Bohlke

Rodolfo Bonatti

Anjan K. Bongoni

Ann Bonner

Mario Bonomini

Ray Borrow

Dorin-Bogdan Borza

Willem Jan W Bos

Bhadran Bose

Joel Bozue

Patrícia C. Braga

Lulu Bravo

Robert Bright

David A. Broniatowski

Klaus Brusgaard

Semra Bulbuloglu

Nicolas Burdin

Nicolas Burdin

Erlina Burhan

Kevin D. Burns

Conor Cahill

Hong Cai

Qiaoting Cai

Murat Çakır

Robert C Albright Jr

Zafer Çalıskan

Sefa Burak Çam

Kathryn Campy

Myrna Candelaria

Mustafa Candemir

Edgar Cano-Europa

Ugur Canpolat

Gerard Cantero-Recasens

Hualin Cao

Jing-Yuan Cao

Wenlong Cao

Yang Cao

Yingshu Cao

Yirui Cao

Alessandro Capitanini

Brenno Cardoso Gomes

John Carlson

Zoccali, Carmine

Rafael Lima Rodrigues De Carvalho

Douglas J. Casa

Sofia A. Casares

Danilo Casimiro

Santos Castañeda

Angel Denisse Castro-Eguiluz

Tiffany N. Caza

Pribil Celine

Anis Chaari

Bryce Chackerian

Miloud Chakit

Ching Ho Chan

Daniel Tak Mao Chan

Gek Cher Chan

Yung Wai Chan

Wiwat Chancharoenthana

Jayanthi Chandar

Saket Singh Chandel

Alexander R Chang

Elizabeth Chang

Jie Chang

Liang Chang

Liyang Chang

Evangelia Charitaki

Antoine Chatrenet

Katrina Chau

Kunal Chaudhary

Chenchit Chayachinda

Charles Chazot

Bingru Chen

Chaojin Chen

Chien-Liang Chen

Haiyong Chen

Hongbo Chen

Jihong Chen

Ji-Yan Chen

Juntao Chen

Liangmei Chen

Ling Chen

Lin H. Chen

Nan Chen

Tianxin Chen

Wangxue Chen

Xiangyu Chen

Xiao-Peng Chen

Yan Chen

Yang Chen

Yingyao Chen

Yuanzhi Chen

Yue Chen

Yung-Hsiang Chen

Zhaowei Chen

Alice Y. Y. Cheng

Hong Cheng

Jizhong Cheng

Heng-Pin Chiang

Kittiphan Chienwichai

Chirawat Chiewchalermsri

Lazar Chisavu

Frederick Nchang Cho

Ana Marli Christovam Sartori

Angel Cid‐Arregui

Secundino Cigarrán Guldris

Aleksandar Cirovic

Madhu Cl

Thomas Clanton

Katherine Clark

Mario Coccia

Camille Cohen

Daniel Cohen

Manuel Colome

Giacomo Colussi

Veronica Cordoba Sanchez

Sarah Cormican

Diana Coronel

Cosmin Cozma

Fabrizio Cristiano

Alessandro Crocoli

Nicolas Cuburu

Fangqiang Cui

Jiong Cui

Tianlei Cui

Ying Cui

Medine Cumhur Cure

Anthony Lawrence Cunningham

Erkan Cüre

Carlo Curina

Salvatore Lucio Cutuli

Anna Cyrek

Szabina Czirok

Kiran Dahiya

Xuan Dai

Maria Aparecida Dalboni

Li Dandan

Ahmed Daoud

Sihem Darouich

Manas Das

Ramtin Dastgir

Manjula Datta

Vinoi George David

Simon J. Davies

Dilip K Deb

Jeroen H. F. De Baaij

Iris De Bruijn

Elizabeth De Gaspari

Annie De Groot

David Degroot

Erik Pieter De Jong

Joris Delanghe

Luis Del Carpio-Orantes

Cristian Delcea

Mark D. Elliott

Giovanni Delogu

Yeliz Demir

Lili Deng

Pengfei Deng

Yang Deng

Aleksandar Denic

Anne-Cornelie De Pont

Aravinda Desilva

Paolo De Simone

Perumal Arumugam Desingu

Matheus De Souza

Sreebhushan Devaraju

Ganesan Dhanasekaran

Moussa Dia

Don J Diamond

Cristiane Dias

Robert Dillman

Antonio Di Lorenzo

Giuseppe Di Martino

Joshua Dinapoli

Gungor Cagdas Dincel

Nan Ding

Runmin Ding

Ying Ding

Dai Xuan Dinh

Charles Diskin

Hana Dobrovolny

Xiangyu Doctor

Matthieu Domenech De Cellès

Bingzi Dong

Boqing Dong

Jie Dong

Laetitia Dou

Yanna Dou

Robert Dragu

Johannes W Drouven

Dorota DrożDż

Weigang Duan

Anila Duni

Ayşen Durak Aslan

Nihal Durmaz

Antoine Dürrbach

İsmail Dursun

Fiona Ecarnot

Raquel Echavarria

Michael Edelstein

Peter J. Eggenhuizen

Maryam Eidi

Laila A. Eissa

Robert Ekart

Ubong S Ekperikpe

Mohamed Ahmed Eladl

Adel Elatreisy

Ahmed Hussieny Elbarbary

Mohamed El-Boshy

Elias John Elenjickal

Mohamed Abdelaziz El-Gamasy

Hesham A Elghoneimy

Daniele Elias

Rosilene M. Elias

Christopher El Mouhayyar

Gehad El Nakib

Heba El Said

Ibrahim El-Serafi

Alia El Shahawy

Magdy El Sharkawy

Antoine Enfrein

Paul Engeroff

Takayuki Enomoto

Ayşegül Erdoğan

Bülent Ergin

Sercan Ergün

Ertugrul Erken

Alparslan Ersoy

Makram Essafi

Kübra Evren

Donald E Wesson

Fabio Fabbian

Peter Fairman

Rui-Feng Fan

Ying Fan

Jie Fang

Ming Fang

Yi-Peng Fang

Mma Faridi

Anahita Fathi

Sarah Faubel

Stephen Fava

Huimei Feng

Quan-Sheng Feng

Xianxuan Feng

Sandro Feriozzi

Leyla Ferlicolak

Anders Fernström

Elen Ferraz Teston

William Fissell

Wayne Fitzgibbon

Adrien Flahault

Alison Footman

Ivo Foppa

Dorota Formanowicz

Marco Franchin

Maria Giovanna Francipane

Richard Franka

Ivo Fridolin

Catherine Fry

Bin Fu

Chuanxi Fu

Xuebin Fu

Flávio Danni Fuchs

Tomoyuki Fujikura

Takuya Fujimaru

Akihiro Fukuda

Seiji Fukumoto

Tibor Fulop

Winston Fung

Liliana Gadola

Daniel Gaffiero

Morgan Gallazzini

Miguel Gallego Munuera

Maurizio Gallieni

Amir Gal-Oz

Ananthakrishnan Ganesan

Bingmiao Gao

Hanchao Gao

Xia Gao

Xuxia Gao

Ya-Bin Gao

Yue Gao

Zhao Gao

Manuel García Cenoz

Andrea Garcia-Lopez

Maha Gasmi

Pragya Gautam Poudel

Nathalie Gayrard

Alfredo G Casanova

Ning Ge

Xin Ge

Xuhua Ge

Yongchun Ge

Nicholas Geard

Martin Gebel

Guido Gembillo

Federica Genovese

Aman George

Gijo George

Jacob George

James F George

Robert M. Geraghty

Alexandre O. Gérard

Michael Germain

Kamel Gharaibeh

Marzieh Saei Ghare Naz

Christoforos Giannaki

Amnon Gil

Catherine Gilchrist

Francesco Giombi

Mohan Giri

Nikolaos Gkiourtzis

Patricia Goentoro

Miguel Angelo Goes

Shoshanna Goldin

Miquel Gomez Umbert

Raul Gomila

Tibor Gondos

Minmin Gong

Nianqiao Gong

Xuezhong Gong

Ioannis Goniotakis

Erdem Gönüllü

Camilo Alberto Gonzalez-Gonzalez

Natarajan Gopalakrishnan

Kamal R Goplani

Andrew Gorringe

Jose Luis Górriz

Hiroyasu Goto

Pavlos Goudas

John Grabenstein

Noble Gracious

David Greenberg

Tom Greene

Charles Grose

John Grosser

Anders Grubb

Menglei Gu

Mingjia Gu

Wan-Jie Gu

Wen Gu

Xiangchen Gu

Wang Guangfei

Huan Gui

Cuma Bulent Gul

Mustafa Güldan

Mustafa Can Guler

Iosif Gulkarov

Emrah Gün

Emrah Gunay

Hongxiong Guo

Qiang Guo

Xiaojiao Guo

Yun Guo

Alok Gupta

Sanjay Gupta

Leonard Gurgaș

Gail Gustavson

Geeta Gyamlani

Elmukhtar Habas

Mehmet Haberal

Charbell Miguel Haddad Kury

Amir Hadi

Gaye Hafez

Sakineh Hajebrahimi

Mohadeseh Haji Abdolvahab

Scott B Halstead

Satoshi Hamada

Itsuki Hamamoto

Yoshifumi Hamasaki

Yuko Hamasaki

Muhamad R. Abdel Hameed

Mehrdad Hamrahian

Huirong Han

Jiahao Han

Jinming Han

Min Han

Weixiao Han

Ramy M Hanna

Bo Terning Hansen

Ashwathnarayana Hanumanthaiah

Jie Hao

Junfeng Hao

Warda Haque

Satoshi Hara

Joshua Hare

Geoffroy Hariri

Agnes Haris

Michiel M. Harmsen

Daniel A. Harris

Jan Miroslav Hartinger

Kotaro Haruhara

Marwa S. Hassan

Denise C Hasson

Ashik Hayat

Mary Hayney

Abu Hazafa

Chiyi He

Junbing He

Qing He

Riming He

Zhaohui He

Matthew K Hensley

Demian Arturo Herrera Morban

Christian Herzog

Javad Heshmati

Elisabeth Hesse

Elisabeth M. Hesse

Samuel N. Heyman

Emma J Heymer

Sumi Hidaka

William Hinton

Yosuke Hirakawa

Swapnil Hiremath

Yuh-Shan Ho

Nick Holford

Hanzhang Hong

Keerati Hongsakul

Taro Horino

Doosadee Hormdee

Keiko Hosohata

Emran Hossain

Jian Hou

Shun-Neng Hsu

Chang Hu

Dongsen Hu

Dongsheng Hu

Haofei Hu

Jianguang Hu

Liang Hu

Linhui Hu

Peng Hu

Qiong-Dan Hu

Shasha Hu

Shuai Hu

Xiao Hu

Yao Hu

Yuxin Hu

Weng Huachun

Bo Huang

Gang Huang

Guangrui Huang

Hongdong Huang

Howard Huang

Huangjingda Huang

Hui Huang

Kaipeng Huang

Kun Huang

Lanting Huang

Mingcheng Huang

Naya Huang

Quanyuan Huang

Suiqing Huang

Wei-Jun Huang

Xianghua Huang

Xiongfeng Huang

Miguel Hueso

Wun Fung Hui

Jim Huleatt

Harvey David Humes

Bengang Huo

Sadique Hussain

Mohamad A. Hussain

Zeb Hussain

David Hutton

Fortunato Iacovelli

Viktoriia Iakovleva

Tatsuyoshi Ikenoue

Hidekazu Ikeuchi

Toshiyuki Imasawa

John Imig

Gokhan Inangil

Tsutomu Inoue

Ashley Irish

Stephanie Irving

Berend Isermann

Tomona Iso

Bátai István

Katsuhiro Ito

Marie Ito

Kazuhiro Itoh

Shahrokh Izadi

Ayesha Jabeen

Ameneh Jafari

Saeideh Jafarinejad-Farsangi

Edgar A Jaimes

Parag N. Jain

Mostafa Jamalan

Karin Agnes Maria Jandeleit-Dahm

Carolina Janovsky

Thabiet Jardine

Ajay Jaryal

J. Ong Jason

Seyed Alireza Javadinia

Tarun Jeloka

Tahmina Jesmin

Niraj Kumar Jha

Pranaw Kumar Jha

Congzhuo Jia

Aimin Jiang

Binshan Jiang

Wuhua Jiang

Xiaowen Jiang

Yanfang Jiang

Zhi-Wei Jiang

Jianghua Zhou Jianghua Zhou

Baihai Jiao

S Jimenez-Jorge

Heng Jin

Hui Jin

Qi Jin

Shi Jin

Nobuhiko Joki

Aaron Jones

Jonny Jonny

Adrien Joseph

Guanqun Ju

Jiang Jun

Anna Kablak-Ziembicka

Ika Kadariswantiningsih

Kaisaierjiang Kadier

Nur-Ayuni Kadir

Wisit Kaewput

Dogan Kahraman

OğUzhan Kahraman

Farzad Kakaei

Andrew Kalra

Yuka Kamijo

Meral Kanbak

Aravind Kancharla

Sinan Kandir

Seok Hui Kang

Yu-Lin Kang

Joshua Kaplan

Ali Veysel Kara

Archana Karadkhele

Paschalis Karakasis

Süleyman Karaköse

Masato Karayama

Asad Karim

Masato Kasahara

Ozgur Kasapcopur

Pisut Katavetin

Mehtap Kavurmaci

Yoshiki Kawamura

Ahmet Kazez

Ben Ke

Guibao Ke

Natasha Kelkar

Mina Kelleni

Elizabeth Kelly

Bryce A. Kerlin

Eric Kerns

Nisha Keshary Bhatta

Christodoulos Keskinis

Kent Kester

Kanwal Khalid

Steve Khalil

Irfanullah Khan

Muhammad Ali Khan

Rafiq Ahmad Khan

Tila Khan

Uzma Basit Khan

Maged Kharouba

Madan Khatiwada

Manoj Khokhar

Dinesh K. Khullar

Ahmet Kasim Kilic

Dana Kim

Dong Ho Kim

Gheun-Ho Kim

Hyunsuk Kim

Yaerim Kim

Yasuyuki Kinjo

Yoshitaka Kinoshita

Eva Kiousi

Attila Kiss

Taito Kitano

Orit Kliuk-Ben Bassat

Satoshi Kobayashi

Shuzo Kobayashi

Gülay Koçak

Christian Koch

Hans Koehnk

Sho Kojima

Gabor Kokeny

Peter Kokol

Jay K. Kolls

Weiyi Kong

Yaozhong Kong

Yonglun Kong

Agni Konitsioti

Abhijit Konnur

Jeroen P. Kooman

Kristina Kopeva

Csaba Kopitko

Jeffrey B. Kopp

Raphaël Kormann

Sradha Kotwal

Christos Kourek

Jesse C. Krakauer

Holly J. Kramer

Chatchai Kreepala

Leonard R. Krilov

Pavla Krizova

Manuel Krone

Abhijit Kshirsagar

Ernest Kuchar

Akihiro Kuma

Ashok Kumar

Deepak Kumar

Naveen Kumar

Prashant Kumar

Nitu Kumari

Liu Kun

Michael Kundi

Chan-Yen Kuo

Ko-Lin Kuo

Akif Kurt

Pervin Ozkan Kurtgoz

Ilhan Kurultak

Pooja Kushwaha

Prit Kusirisin

Andrzej Kutarski

Yury Kuzmichev

Mike Yat Wah Kwan

Jia Liang Kwek

Hyunwook Kwon

Osun Kwon

Ayşenur Paç Kısaarslan

Hakan Kısaoğlu

Majid Laassri

Kaice Lafavers

Kamel Laghmani

Alexander Lai

Xueli Lai

Daniel Landau

Nuttasith Larpparisuth

Anders Larsson

Abdul Latif

Renat Latipov

Yu Lung Lau

Jeffrey Laurence

Patrizia Laurenti

Pascal Lavoie

Miltos Lazarides

Miltos K. Lazarides

Eduardo Cesar Lazcano Ponce

David Leavesley

Eleanor D Lederer

Jin Lee

Juhan Lee

Su Mi Lee

Yeonggeul Lee

Chong Lei

Li Lei

Wenhui Lei

Vasileios Lekakis

Pui-Ying Leong

Ann Levin

Myron J. Levin

Yves Levy

Aiqing Li

Changqun Li

Changying Li

Delun Li

Duo Li

Hao Li

Hong-Bao Li

Ji-An Li

Jianbo Li

Jingkuo Li

Jiongming Li

Junhua Li

Le Li

Longkai Li

Min Li

Ming Li

Mintao Li

Qihan Li

Qiuyang Li

Sheyu Li

Wen-Xiong Li

Xianzhi Li

Xiaobing Li

Xiaoyu Li

Xinli Li

Xudong Li

Xudong Li

Yang Li

Yi-Chen Li

Yifu Li

Yucong Li

Zi Li

Bo Liang

Haidong Liang

Hao Liang

Hongwei Liang

Min Liang

Qiaojing Liang

Yan Liang

Zehuan Liao

Cynthia Ciwei Lim

Dwee Wee Lim

Lee-Moay Lim

Chih-Feng Lin

Chung-Ying Lin

Hugo You-Hsien Lin

Ming-Yen Lin

Qisheng Lin

Wei Lin

Xiaomeng Lin

Xinghui Lin

Yanjuan Lin

Zishan Lin

Megan Lindley

Chen Ling

Chan Liu

Chan-Min Liu

Fengxun Liu

Ge Liu

Guangjin Liu

Hailei Liu

Hongxing Liu

Huafeng Liu

Jian-Jun Liu

Jin Liu

Juan Liu

Jun Liu

Mei Ying Liu

Meng Liu

Qi Liu

Tao Liu

Tongqiang Liu

Xiang Liu

Xiangliang Liu

Xiaoping Liu

Xing Liu

Xingzheng Liu

Xingzi Liu

Xiu-Juan Liu

Xueen Liu

Xuyan Liu

Ya-Fei Liu

Yang Liu

Yingwu Liu

Yingying Liu

Yue Liu

Yuxiang Liu

Yuxue Liu

Zhao Liu

Zhenyu Liu

Navin Kumar Loganadan

Gianmarco Lombardi

Jun Long

Helen C Looker

Ángel Arturo López-González

Paul Loubet

Charalampos Loutradis

Siyuan Lu

Wei Lu

Xinjun Lu

Yihan Lu

Zhonghua Lu

Lin Luan

Ning Luan

Zhi-Lin Luan

Roxana Liana Lucaciu

Leandro Lucca

Marko Lucijanic

Brandon Lucke-Wold

David J Lundy

Jan Lunemann

Changfang Luo

Laimin Luo

Cindy Luongo

Daoyuan Lv

Min Lv

Swathi M

Bowei Ma

Dong Hong Ma

Huabin Ma

Ke Ma

Xiao Ma

Iain C. Macdougall

Alisson Machado

Masaki Machida

Yujiro Maeoka

Roshan Kumar Mahat

Cedric Mahe

Syed Mahmood

Yanpei Mai

Olusesan Makinde

Mai M. Malek

Kunal Malhotra

Jan Malik

Benjamin Malo

Grace Mambula

Salvatore Mandolfo

Aysun ManisalıGil

Amin Mansoori

Juliana Mansur

Mukta Mantan

Renato Mantegazza

Jian-Hua Mao

Wenjun Mao

Ilaria Mariani

Ollyvia Freeska Dwi Marta

Maximiliano Martin

Laisel Martinez

Nataša Marčun Varda

Lucia Masiero

Joel Maslow

Francesco Massari

Yoshio Masuda

Georgie Mathew

Gerry George Mathew

Roy O Mathew

Nor Aziyah Mat Rahim

Joseph Mattana

Edison Johannes Mavundza

Muniaraj Mayilsamy

Marilda Mazzali

Alessio Mazzieri

Jolanta Małyszko

Lisa Mchugh

Graham Mcilroy

Gary R. R. Mclean

Lawrence P Mcmahon

Guruprasad R. R. Medigeshi

Irsida Mehmeti

Clemens M. Meier

John Mellas

Marcela Mendes

Juan Meng

Giovanni Merlino

A. Lianne Messchendorp

Laurent Metzinger

Michelle Meyer

Nadia M. Hamdy

Nelson L Michael

Ryoma Michishita

Adam C Midgley

Renzo Mignani

Irena Milaniak

Marija Milinkovic

Aaron W. Miller

Satoshi Minami

Gourav Mishra

Barbara M Misof

Constance A. Mitchell

Palash Mitra

Karina Takesaki Miyaji

Tetsu Miyamoto

Akio Miyasaka

Hoda Mohamed

Tarek Mohamed

Ran Mohan

Ranjan Kumar Mohapatra

Reza Mohebbati

Lenny Moise

CC Mok

Yousef Mokary

Tahmineh Mokhtari

Anissa Moktefi

Miklos Z Molnar

Donald A Molony

Fabrizio Monaco

Pasquale Mone

Norihito Moniwa

Marc R Moon

Seyed Moosavi

Javier Morales

Lázaro Moreira Marques Neto

Stefano Moretto

Yutaro Mori

Diego Moriconi

Daisuke Morinaga

Naoki Morito

Daniel L. Moss

Ahmad Movahedpour

Shahrul Mt-Isa

Muhammed Mubarak

María Jimena Muciño Bermejo

Rudraksh Mukherjee

Dhanunjay Mukhi

Ryan Mullane

Minoru Murakami

Karumathil M Murali

Anusha Muralidhar

Efrén Murillo-Zamora

Manuel Muro

Harini N

Ning Na

Kiyotaka Nagahama

Misako Nagasaka

Shokichi Naito

Rami Najjar

Georgina Nakafero

Akihiro Nakamura

Michio Nakamura

Shingo Nakayama

Srinivas Nanduri

Hamano Naoto

Tarun Narang

Matteo Nardin

Fateme Nateghi Haredasht

Aya Nawata

Allen Nazareno

Valantine Ndze

Julia Neufeind

Jack Kit-Chung Ng

Qin Xiang Ng

Yue-Harn Ng

Ntombenhle J Ngcobo

Nga Nguyen

Timothy Nguyen

Hai Nguyen-Tran

Dan Alexandru Niculescu

Ping Nie

Ole Haagen Nielsen

Sebastian Nielsen

Lovelesh Nigam

Guijun Ning

Liping Ning

Meng Ning

Rajkishor Nishad

John Njuma Libwea

Aliocha Nkodila

Chimeremma Nnadi

Surapon Nochaiwong

Ryunosuke Noda

Sanjeev Noel

Mahin Nomali

Alberto Nordmann-Gomes

Joëlle L. Nortier

Takehiko Oami

Yoshitsugu Obi

Matthias Ocker

Patrick O’Connell

Takashi Oda

James Odum

Ifeanyichukwu Anthony Ogueji

Olorunfemi Ogundele

Dong-Jin Oh

Masahiro Okabe

Keisuke Okamoto

Ogochukwu Okoye

Timothy Olusegun Olanrewaju

Victoria Olarewaju

Sonja Oleary

Agnieszka Olejnik

Piotr Olejnik

Ole F. Olesen

Erika Melendez Oliva

Bukola Opeyemi Oluwarinde

Esteban Ortiz-Prado

Nosayaba Osazuwa-Peters

Nicholas Osborne

Megumi Oshima

Yasser Osman

Jelena Nesovic Ostojic

Naoyuki Otani

Yanqiu Ou

Connie P. C. Ow

Oladayo A Oyebanji

Ahmet Oz

Manisha J. Oza

LaşIn Özbek

Aydan Akyüz Özdemir

Mustafa Ozdemir

Sare Gülfem Özlü

Hayrettin Ozturk

Ilyas Öztürk

Savaş Öztürk

Lukas Page

Sonali Palkar

Annapina Palmieri

Binbin Pan

Chun Pan

Yangbin Pan

Yu Pan

Angeliki Panagiotou

Yuanita Panma

Ruchir Pansuriya

Vassiliki Papaevangelou

Nikolaos Papanas

Kutae Park

Meyeon Park

Woo Yeong Park

Amit Pasari

Anil Kumar Pasupulati

Dhaval Patel

Tejas K. Patel

Rolando Paternina-De La Ossa

Brandon Patterson

Robert Paulino-Ramirez

Alex Pauvolid-Corrêa

Nikola M. Pavlović

Saime Paydas

Mark Peeples

Huaying Pei

Hui Peng

Yonghan Peng

Jennifer Pereira

Luciano Pereira

Susan Pereira Ribeiro

Antonino Perino

Amy Perry

Diogo B Peruchetti

Licia Peruzzi

Ákos Géza Pethő

Marek Petrá

Tadej Petreski

Giorgina Barbara Piccoli

Nicola Pirozzi

Nuntawan Piyaphanee

Mirha Pjanic

Stanley Plokin

Shirley Pollack

Rafael Ponikvar

Otilia Popa

Steven A. Porcelli

Attur Ravindra Prabhu

Narayan Prasad

Jeffrey Presneill

Susan E. Prockop

Menno Pruijm

Dominik Pruski

Dechu P. Puliyanda

Patrick Pun

Heider Qassam

Xingshun Qi

Yi-Jun Qi

Xi Qiao

Shu-Lan Qin

Chao Qiu

Ming-Zhu Qiu

Zongyao Qiu

Shujuan Qu

Jean-Sebastien Rachoin

Trina Racine

Omar Ragy

Hamid-Reza Rahimi

Mohamed Rahouma

Pradeep Rai

Jasmin Raja

Shahzad Gull Raja

Raja Ramachandran

Indu Ramachandra Rao

Abeer Ramadan

Kumaresan Ramanathan

Jesus Manolo Ramos-Gordillo

Shira Ramot

Harish Rangareddy

Xiangrong Rao

Milan Raska

Matteo Ratti

Lisa Mary Raven

Hugh Rayner

Maycon Moura Reboredo

Ahmed Reda

Jeff Reeve

Francesco Reggiani

Christine Rehm

Željko Reiner

Jochen Reiser

Thomas Remer

Andrea Remuzzi

Giuseppe Remuzzi

Guangqin Ren

Huiwen Ren

Yan Ren

Claude J. Renaud

Sasirekha Rengaraj

Bernhard Resch

Rajesh Reviewer

Oleksa G. Rewa

Lotfollah Rezagholizadeh

Omid Rezahosseini

Mohammad Rezapour

Sharon D Ricardo

Luisa Ricciardi

Matteo Riccò

Luis Rico

Nicasio Rini

Syed Faheem Askari Rizvi

Michelle L. Robbin

Ramon Roca-Tey

Joao Rocha-Neves

Michaël Rochoy

Lance E. Rodewald

Emilio Rodrigo

Vinayak Rohan

Mohsen Rokni

Giandomenico Roviello

Gautam R. Shroff

Giordano Ruggeri

Rohit Ruhal

Aleksandr Rumyansev

Jakub Ruszkowski

Ekaterina Ryabchevskaya

Alicja Rydzewska-Rosolowska

Aleksandra Rymarz

Saranyadevi S

Jeann Luccas Sabino-Carvalho

Gianmarco Sabiu

Mahmoud S. Sabra

Robert Safirstein

Jeffrey Safrit

Sukumar Saha

Serap Sahin-Bolukbasi

Muthana Al Sahlawi

Tuncay Sahutoglu

Takao Saito

Yosuke Saka

Ken Sakai

Imene Bouchra Sakhi

Giorgos Sakkas

Mahmoud Salam

Asmaa Salah Eldin Mohamed Saleh

Sohail Abdul Salim

Beatriz Sánchez Moreno

Andrea Sánchez-Navarro

Donato Santovito

Gaetallo Santulli

Dwi Cahyani Ratna Sari

Takayoshi Sasako,

Sandeep Sasidharan

Emi Sato

Shuzo Sato

Michihiro Satoh

Shailendra K Saxena

Carlos Scartascini

Enrico Schalk

Jorg Schelling

Daniel Schneditz

Kirsten Schneider-Ohrum

Florian Schödel

Jemima K. Scott

Sophie Sears

Joel Segel

Cristian Segura

Vishaldeep Sekhon

Nedim Selcuk

Jothiraj Selvaraj

Ismail Selvi

Marharyta Semenikhina

Anirban Sengupta

Concetto Sessa

Didik Setiawan

Ankur Shah

Fatemeh Shahbazi

Robert Shahverdyan

Itamar Shalit

Yi Shao

Wang Shaoqing

Usama Sharaf El Din

Jana Shaw

Ryan Sheikh

Feng Shen

Liming Shen

Qian Shen

Srinivas Vinayak Shenoy

Vicky Sheppeard

Jia Shi

Rui Shi

Wen Shi

Yiqin Shi

Yue Shi

Hisato Shima

Satoshi Shimada

Yutaka Shimazu

Michelle Shin

Li Shu

Yue Shu

Evgeny Shutov

Saulat Siddique

Tabo Sikaneta

Jeffrey Silberzweig

Ricardo Silvariño

John J. Sim

Mariadelina Simeoni

Eric A. Singer

Mahmoud Singer

Alok Singh Singh

Desh Deepak Singh

Manjinder Singh

Pawan Kumar Singh

Prabhat Singh

Vishal Singh

Waryaam Singh

Rano Sinuraya

Meghan E Sise

Ramakrishnan Sitaraman

Judith Smith

Igor Smolenov

Amin Soliman

Karim Soliman

Saeed Soliman

Laurie R. Solomon

Leilah Sonday

Jiayu Song

Jinfang Song

Kallendrusch Sonja

Herbert Sophia M C

Sveinung Wergeland Sørbye

Alieu Sowe

Andrea Spasiano

Goce Spasovski

Armand Sprecher

Jordan Stanford

Aristeidis Stavroulopoulos

Ladislav Stepanek

Natalia Stepanova

Dominik Steubl

Kostas Stylianou

Guobin Su

Jiaming Su

Qinyue Su

Kotaro Suemitsu

Long Sui

Zhou Suizan

Mahaboob Khan Sulaiman

John Sullivan

Chuan-Chuan Sun

Jin-Peng Sun

Lulu Sun

Qi Ce Sun

Zeguo Sun

Zhaoxing Sun

Choa Sung

Junne-Ming Sung

Siddharth Sunilkumar

Hani Susianti

Éva Szabó

Laszlo Szabo

Balazs Szamosfalvi

Reza Tabrizi

Silvio Tafuri

Maxime Taghavi

Saeed Taheri

Amir Taherkhani

Ming Tan

Renzhi Tan

Rui-Zhi Tan

Ying Tan

Hiroki Tanabe

Hiroshi Tanaka

Kenichi Tanaka

Sevgin Taner

Chengchun Tang

Hailin Tang

Haoxian Tang

Ju-Yu Tang

Lili Tang

Shuifu Tang

Wen Tang

Xinfang Tang

Yuyan Tang

Zhaohui Tang

Elliot Koranteng Tannor

Tawee Tanvetyanon

Mihály Tapolyai

Surjit Tarafdar

Philip Tarr

James Tartaglia

Amir Hossein Tashakor

Hamid Tayebi-Khosroshahi

Maxime Teisseyre

J. Pedro Teixeira

Esra TekİN

Xu Teng

Jonne Terstappen

Vladimír Tesař

Wubshet Hailu Tesfaye

Bijin Thajudeen

Juthika Thaker

Peerapat Thanapongsatorn

Pugazhenthan Thangaraju

Damien Thibaudin

Diraviyam Thirumalai

Stephen Thomas

Edward Thommes

Subhash Thuluva

Huidi Tian

Na Tian

Pengchao Tian

Ye Tian

Emma Tillman

Juan Miguel Díaz Tocados

Kacper Toczylowski

Burkhard Toenshoff

Gabriele Toietta

Filip Tomović

Francesco Tondolo

Fei Tong

A. Toniolo

Senol Tonyali

Kumiko Torisu

Ryma Toumi

Jonathan N. Townend

Huseyin Toz

Bryony Treston

Gaurav Tripathi

Ming-Tsun Tsai

Wei-Cheng Tseng

Makoto Tsujita

Ikuo Tsunoda

Chen Tu

Tugbabedir Tugbabedir

Deniz Tuncer

Tao-Hsin Tung

David J Tunnicliffe

Faruk Hilmi Turgut

Rachel Turn

Daniel Turudic

Yoshifumi Ubara

Kiyotaka Uchiyama

Genta Uehara

Mugen Ujiie

KadİR Ulu

George Umemoto

Anetta Undas

Velid Unsal

Sean Upshaw

Matthew W. Urban

Luciano Urdinez

Sadettin Uslu

Arife Uslu Gokceoglu

Len A Usvyat

Takashi Uzu

Sakshi Vaishnav

Anthony Valeri

Kalliopi Vallianou

Wim Vandenberghe

H Rogier Van Doorn

Simon D Van Haren

Aruna Vanikar

Hester Van Zeeburg

Armando Vargas-Palacios

Vipin M. Vashishtha

Roberto I Vazquez-Padron

Jorge Vega

Xavier Vela Parada

Victoria Velarde

Andrea Aida Velasco Medina

Arzu Velioglu

Himanshu Verma

Giuseppe Vezzoli

Pedro Rafael Vieira De Oliveira Salerno

Davide Viggiano

Madhusudan Vijayan

Marina Vivarelli

Simonetta Viviani

Giau Vo

Giau Van Vo

Gianluca Voglino

Eva Vonbrunn

Thilo Von Groote

Bozidar Vujicic

Yukihiro Wada

Rowan Walker

Jianxin Wan

Li Wan

Shanshan Wan

Ori Wand

Baodong Wang

Bingqiao Wang

Dawei Wang

De-Guang Wang

Dong Wang

Fang Wang

Feng Wang

Fengying Wang

Hao Wang

Heng Wang

Hongliang Wang

Huaiyu Wang

Huiming Wang

Jianwen Wang

Ji-Guang Wang

Jingyu Wang

Kaihong Wang

Li Wang

Liang Wang

Lulu Wang

Luoyi Wang

Mengting Wang

Ming Wang

Peng Wang

Ran Wang

Sheng-Fan Wang

Shuai Wang

Sophia S. Wang

Tingting Wang

Tun Wang

Wei Wang

Weiming Wang

Weiyi Wang

Xinyao Wang

Yajuan Wang

Yilin Wang

Yiqin Wang

Yongfang Wang

Zhenhua Wang

Zhenwei Wang

Zhilian Wang

Ziheng Wang

Zixin Wang

Zunsong Wang

Brian Ward

Leigh C. Ward

Echo Warner

William Warren

Hirofumi Watanabe

Chengguo Wei

Jianxin Wei

Kai-Che Wei

Lixin Wei

Weisheng Feng

Qiang Wen

Scott E. E. Wenderfer

Zebin Weng

Rehab H. Werida

Solange Whegang Youdom

Theresa L. Whiteside

Tri Wibawa

Ahmedz Widiasta

Alice Wiedeman

Scott Maxwell Wilson

Chew Ming Wong

Limy Wong

Miu-Ling Wong

Graham Woodrow

Minichil Chanie Worku

Viktoria Woronik

Buyun Wu

Chung-Kuan Wu

Da-Wei Wu

Dong-Dong Wu

Haiyang Wu

Jun Wu

Junnan Wu

Lingling Wu

Sensen Wu

Taijung Wu

Yong Wu

Zheng-Hao Wu

Zhong Wu

Zhongkai Wu

David Wullimann

Xiaojun Xia

Fangfang Xiang

Yi Xiang

Li Xiao

Qiong Xiao

Xiaohuan Xiaohuan

Danshu Xie

Dengpiao Xie

Jingzhi Xie

Liangdi Xie

Xiang Xie

Yan Xing

Zailing Xing

Erfeng Xiong

Yunhe Xiong

Zhile Xiong

An P. Xu

Binyue Xu

Bo Xu

Guang Xu

Lingyi Xu

Long Xu

Shi-Wen Xu

Tao Xu

Wang Xu

Yan Xu

Wei Xu

Nadir Yalçın

Shunsuke Yamada

Shintaro Yamaguchi

Ryo Yamamoto

Junji Yamauchi

Tomohiko Yamazaki

Bingyu Yan

Jianyun Yan

Jiayi Yan

Liang-Jun Yan

Ming Yan

Xianglei Yan

Changyuan Yang

Guang Yang

Guoping Yang

Junzheng Yang

Li Yang

Min Yang

Ming Yang

Qiao Yang

Wen-Ling Yang

Xiaohan Yang

Xiao-Wei Yang

Yong Yang

Yunwen Yang

Zhou Yang

Congcong Yao

Lu Yao

Xingchen Yao

Yit-Sheung Yap

Muhammad Osama Yaseen

Barbara Yawn

PıNar YazıCı Özkaya

Jiakai Ye

Nan Ye

Yu-Wen Yeh

Lenar Yessayan

Gürsel Yildiz

Akar Yilmaz

Ebru Yilmaz

Liang Ying

Tadashi Yoshida

Jun Yoshino

Megan Young

Zahra Yousefi

Gamal Youssef

Chen Yu

Guizhen Yu

Nannan Yu

Shengyou Yu

Xiangyou Yu

Xiaojuan Yu

Yang Yu

Zihua Yu

Jiangzi Yuan

Sibel Yucel Kocak

Mehtap Yüksel Egrilmez

Anand Yuvaraj

Tolga YıLdıRıM

Rajeev Zachariah Kompithra

Gunther Zahner

Insha Zahoor

Mohamed H. Zahran

Izabela Zakrocka

Sheikh Farzana Zaman

Tobia Zanotto

Michał Zarobkiewicz

Michał Zawistowski

Bahaa Zayed

Dianjie Zeng

Siyao Zeng

Xianchun Zeng

Xianguo Zeng

Zhihe Zeng

Chun-Juan Zhai

Shen Zhan

Ya Zhan

Ao Zhang

Bo Zhang

Chen Zhang

Chong Zhang

Dongliang Zhang

Dongliang Zhang

Guangyuan Zhang

Hongliang Zhang

Jiahe Zhang

Jinghua Zhang

Kang Zhang

Le Zhang

Lei Zhang

Longye Zhang

Minmin Zhang

Pei Zhang

Qin-Hong Zhang

Tao Zhang

Tian Zhang

Wang Zhang

Xiao Zhang

Yimin Zhang

Yin-Xi Zhang

Yong Zhang

Yufei Zhang

Yu-Han Zhang

Zhiqiang Zhang

Zongde Zhang,

Chuanyan Zhao

Guangzu Zhao

Hong Zhao

Huiping Zhao

Jianhui Zhao

Li Zhao

Limei Zhao

Long Zhao

Peng-Yue Zhao

Ping Zhao

Xiujuan Zhao

Yanan Zhao

Yanyan Zhao

Yuliang Zhao

Zhan-Zheng Zhao

Luo Zhen

Guoping Zheng

Liang Zheng

Shutao Zheng

Weibin Zheng

Xinglong Zheng

Xixi Zheng

Huang Zhenyu

Wenqiang Zhi

Fang Zhong

Ming Zhong

Zhengxia Zhong

Changcheng Zhou

Dan Zhou

Hua Zhou

Iris Y Zhou

Jiayi Zhou

Min Zhou

Nan Zhou

Saijun Zhou

Yi-Hua Zhou

Zhuoming Zhou

Jiefu Zhu

Jiemin Zhu

Jinzhou Zhu

Luwen Zhu

Nan Zhu

Tao Zhu

Xu Zhu

Ye Zhu

Yenan Zhu

Xing-Xing Zhuang

Whitney Ziegenhorn

Chen Zijin

Liu Zikang

Edward Zimbudzi

Oren Ziv

Carmine Zoccali

Federica Zotta

Yuanxia Zou

Melisa ŞAhin Tekin

KazıM ŞAhİN

Mustafa şAhin

